# A metaphysical account of agency for technology governance

**DOI:** 10.1007/s00146-024-01941-z

**Published:** 2024-04-21

**Authors:** Sadjad Soltanzadeh

**Affiliations:** University of Amsterdam, Amsterdam, Netherlands

**Keywords:** Capacity-based theories of agency, Agency as impact, Channels of impact, Design and regulation, Human-machine interaction

## Abstract

The way in which agency is conceptualised has implications for understanding human–machine interactions and the governance of technology, especially artificial intelligence (AI) systems. Traditionally, agency is conceptualised as a capacity, defined by intrinsic properties, such as cognitive or volitional facilities. I argue that the capacity-based account of agency is inadequate to explain the dynamics of human–machine interactions and guide technology governance. Instead, I propose to conceptualise agency as impact. Agents as impactful entities can be identified at different *levels*: from the low level of individual entities to the high level of complex socio-technical systems. Entities can impact their surroundings through different *channels*, and more influential channels of impact lead to higher *degrees* of agency. Technology governance must take into account different channels of impact in the contexts of use, design and regulation.

## Introduction

What assumptions about agency underpin current debates on the governance of technology? Are these assumptions tenable? And if not, what alternative account of agency can be employed for these purposes?

An argument has been recurrently used in various proposals and frameworks for technology governance and particularly the governance of artificial intelligence (AI) systems. The argument is that some form of human presence or involvement should be a necessary requirement for morally permissible and legally compliant performance of these systems. Hence, ideas such as human oversight, human control, human-in-the-loop and human-on-the-loop have been proposed to conceptualise the relationship between humans and machines and put this argument into practice. These ideas can be found in official policy documents, such as European Commission’s Ethics Guidelines for Trustworthy AI (2019, 16), EU AI Act (2021, Article 14) and Principles for the Ethical Use of Artificial Intelligence in the United Nations System (2022, 4) as well as in academic debates and civil society recommendations (Asaro 2012; Cavalcante Siebert et al. 2023; Human Rights Watch 2016; Mecacci and Santoni de Sio 2020; Roff and Moyes 2016; Santoni de Sio and van den Hoven 2018; Sharkey 2014; Veluwenkamp 2022; Wagner 2014). 

There is an underlying assumption about agency in this recurring argument. And while official policy documents do not engage in detailed clarifications and their conceptual backgrounds remain open to interpretation, academic and civil society contributions are more clear about their theoretical assumptions. Agents, specifically in these debates, humans, are understood as entities that possess capacities for rational thinking, contextual understanding and value judgement. The presence and involvement of humans with such capacities is argued to contribute to the morally permissible and legally compliant operation of technical systems.

The historical roots of agency as a capacity, often exclusive to humans, can be traced back to Kant’s philosophy and his idea of agents as rational and autonomous beings. Kant’s critical philosophy initiated research programmes in the fields of philosophy of mind and moral philosophy that seek to conceptualise the perceived uniqueness of the human mind, on the one hand, and the rights and responsibilities that stem from this uniqueness, on the other.

Many conceptual contributions to the governance of AI systems also rely on this philosophical tradition. This includes the notion of ‘meaningful human control’ that has been operationalised through classical theories in the philosophy of action (Santoni de Sio and van den Hoven 2018). Central to this notion is the significant role that human normative reasoning should play in the operation of AI-based autonomous systems (Veluwenkamp 2022). Others have used this notion to bridge the gap between philosophical theory and engineering practice (Cavalcante Siebert et al. 2023) and translated the conditions for meaningful human control into design requirements (Mecacci and Santoni de Sio 2020).

My analysis here is not limited to autonomous systems, which have been the main focus of many AI governance ideas, including meaningful human control (Mecacci and Santoni de Sio 2020, 109). I am also not concerned with a specific type of technologies. I maintain a level of generality and concern myself with a broad range of technical systems, including non-digital technologies as well as AI-enabled ones. I argue that intricacies of human-technology relationships require a dynamic understanding of agency and cannot be explained through a capacity-based approach.

In the next two sections, I give an overview of the capacity-based approach and argue how its metaphysical characteristics make it inadequate to serve the purposes of explaining human–machine interactions and supporting technology governance. In response, in the following two sections, I introduce the relational approach and develop the account of ‘agency as impact’ which can serve these purposes.

The fact that I engage in a purposeful reconceptualisation of agency reflects my general philosophical attitude. I believe that metaphysical categories—similar to material objects—can be useful for some purposes and not so useful for others (Soltanzadeh 2019). So, by proposing a new account of agency, my aim is not to discover the universal truth about agency or argue for an overarching definition that should be adopted by different disciplines and for various purposes. Instead, in the vein of what has been recently referred to as the ‘conceptual engineering method’, I am motivated by explanatory and pragmatic considerations (Henne and Huetter-Almerigi 2022). I am interested in a conceptualisation of agency that (1) is suitable for explaining human–machine interactions, and accordingly, (2) can provide a metaphysical underpinning for technology governance. My arguments are primarily metaphysical as they are based on the examination of intrinsic and relational properties that can lead to the attribution of agency.

The most significant contributions of the paper are in the final two sections that introduce ‘levels of agency’ and ‘channels of impact’ in order to examine ‘degrees of agency’. Through the notion of channels of impact, I show that there are different ways in which an entity can exercise its agency. These include goal setting, sensation, evaluation, action, design and regulation. Channels of impact do not necessarily lead to the same degrees of agency, and the hierarchies of the channels of impact can vary in different contexts.

## The capacity-based account of agency

Agency is a foundational concept. We do not necessarily need it to live our everyday lives. But we can utilise it to provide a deeper explanation of social events or justification of normative statements and theories. But how is agency defined? And what makes something an agent?

Traditionally, agency is approached as a capacity, defined by intrinsic properties, such as cognitive or volitional facilities. This approach towards agency can be referred to as the *capacity-based* approach. The capacity-based *approach* has led to various capacity-based *theories* of agency. Capacity-based theories differ in what they regard as *agential capacities*, i.e. properties that are considered essential for agency and delineate agents from non-agents.

Most capacity-based theories are humanistic and individualistic: they define ‘agential capacities’ in such ways that only individual humans can satisfy agency conditions. For example, according to what Schlosser (2019) refers to as the ‘standard notion of agency’, agency requires the capacity for intentional mental states and performing intentional actions; capacities that are traditionally treated by many philosophers of mind as distinctive of individual humans.

The capacity-based approach has also influenced the research into non-individualistic and non-humanistic notions of agency. For instance, Erskine (2001) and List and Pettit (2011), argue that states and corporates also belong to the category of agents as they have ‘agential capacities’ for deliberation and intentional action. Barandiaran et al. (2009) argue that agency, in its minimal sense, requires capacities for goal-directed behaviour which can be exhibited by simple organisms such as bacteria. In philosophy and ethics of technology, too, many discussions around the topic of agency follow the capacity-based approach (Brey 2014; Johnson and Powers 2008; Nyholm 2021; Véliz 2021). Most philosophers argue that at least the current generation of technologies do not have ‘agential capacities’. However, others, such as Floridi and Sanders (2004) or Hildebrandt (2016) define agency based on a set of capacities that machines can also possess. But in any case, regardless of how ‘agential capacities’ are defined, in all capacity-based theories what distinguishes an agent from a non-agent is what agents intrinsically possess.

In the capacity-based approach, once an entity (in the broad philosophical sense of the term, referring to any material or abstract object or system of any size or degree of complexity) is categorised as an agent, its agential status becomes timeless, universal and mind-independent. In this approach, agency is not defined based on people’s perceptions, beliefs or practices. Whether and how agents interact with their surroundings are considered irrelevant for the determination of agency.

Although capacity-based theories define agency based on intrinsic properties of entities rather than their interactions with their surroundings, some links can be drawn between capacities of an entity (be it an agent or non-agent) and the way the entity interacts with its surroundings. Entities’ capacities influence their functions and the type of interactions that they can have (Cummins 1975; Kroes 2012). A credit card, for example, does not have the capacity to hold water, but it has the capacity to tighten some screws. Humans do not have the capacity to fly, but most of them have the capacity to ride a bicycle.

Some capacities of an entity may frequently manifest themselves in the entity’s interactions with other entities, while some others may not actualise and remain as potentials. In the capacity-based approach, agency is defined by the potentials of entities. For example, categorising an entity as an agent may imply that it has the capacity to interact with the world, say, by recommending chess moves, making military decisions or performing a medical diagnostic test. But this definition speaks only to the potential of the entity and does not consider the extent to which that entity’s capacities to recommend chess moves, make military decisions or perform a medical diagnostic test are realised. Regardless of the entity’s actual interactions, its status as an agent (or non-agent) does not change.

The capacity-based approach has its own merits, particularly in the context of interpersonal ethics and substantive accounts of human rights. Griffin (2009), for example, has developed a theory in which all humans have an intrinsic capacity for agency, and the function of human rights is to respect and protect this capacity. In this view, it is irrelevant which social class a person belongs to or whether they are a slave or a feudal lord. What matters in the determination of agency, and hence, in the recognition of human rights, is the capacities that agents (here, humans) should universally possess. So, the capacity-based approach is suitable to conceptualise rights-holders and provide reasons for universal moral duties.

## Inadequacies of the capacity-based approach

The capacity-based account of agency is unfit to explain the dynamics of human–machine interactions and cannot provide a suitable theoretical foundation for technology governance. This is for several reasons.

### ‘Agential capacities’ can be emergent properties

First, in many cases, ‘agential capacities’ cannot be solely attributed to individual human or non-human entities; they can only be attributed to human-technology *relationships*. Technologies such as dice, maps, radar systems and ultrasound technologies contribute to the creation of cognition, decision-making and intentional action (Clark and Chalmers 1998; Kirsh and Maglio 1994; Norman 1991; Sutton 2008; Verbeek 2011). When mental processes are extended to cognitive objects, humans and technologies co-create cognitive action (Rammert 2008). In other words, some attributes that are meant to grant humans unique agential status become the products of human-technology relationships.

Actions and decisions that emerge from human-technology relationships reveal a shortcoming of capacity-based governance ideas, such as the notion of ‘meaningful human control’ in terms of ‘traceability’ of actions and decisions. Santoni de Sio and van den Hoven (2018) build up on moral philosophies of Fischer and Ravizza (1998) to develop an account of meaningful human control, with ‘traceability’ being one of the two conditions for human control. According to this condition, any autonomous system used in sensitive domains, such as military operations, ‘should be designed in such a way as to grant the possibility to always trace back the outcome of its operations to at least one human along the chain of design and operation’ (Santoni de Sio and van den Hoven 2018, 1). This condition can be applied to cases where humans and machines are functionally independent of each other. However, while meaningful human control may be suitable to guide the governance of *autonomous* systems, human–machine interactions are more complex and involve emerging behaviours that cannot be explained by isolated capacities of humans or machines. In cases where actions and decisions emerge from human–technology interactions, the outcome of operations cannot be traced back to individual human or non-human entities.

Some have appealed to the complexity and emergent properties of human–technology interactions to show the inadequacies of the idea of meaningful human control even for the governance of *autonomous* systems. As discussed by Boutin and Woodcock in the context of autonomous weapon systems, current approaches to meaningful human control often fail to recognize that ‘human–machine relationships and military processes are complex, distributed, intermediated, and multidimensional’ (Boutin and Woodcock 2023, 9–10). The point is that autonomous systems are parts of socio-technical systems, and their operation does not happen in a vacuum. Unless autonomous systems reach the level of artificial general intelligence, they will be embedded in broader social contexts involving the users and operators of these systems. The human operator, expected to exercise meaningful control over autonomous systems, is likely to be surrounded by many technologies. And these technologies would hamper the ‘agency and decision-making capacities’ of the operator, which in turn ‘complicate aspirations of human control’ (Schwarz 2021, 55).

Similar points can be made about the second condition of meaningful human control, i.e. ‘the tracking condition’. According to the tracking condition, ‘the system should be able to respond to both the relevant moral reasons of the humans designing and deploying the system and the relevant facts in the environment in which the system operates’ (Santoni de Sio and van den Hoven 2018, 1). An important phrase in the tracking condition is ‘human reasons’ (Veluwenkamp 2022), which is developed through a rationalist understanding of human behaviour (Mecacci and Santoni de Sio 2020, 107–8). It is rooted in an analysis of reasons and actions derived from classical works of Anscombe (1957) and Raz (1975) in the fields of philosophy of mind and philosophy of action. These works were developed prior to the realisation that technologies can play significant roles in the performance of cognitive actions, and consequently, on the exercise of reasoning. They do not engage with the idea that practical reasoning has multiple cognitive ingredients (including gathering sensory inputs, processing and evaluating the inputs, and forming intentions to act upon the world based on the current inputs and memories of past events and actions), which can be extended to and embodied by cognitive objects. Thus, apart from holding a humanist conception of cognition, traditional philosophies of mind also assume an instrumentalist view of technology. In instrumentalist views, technologies are approached as mere tools, and their impact on human reasoning and agency is overlooked. The tracking condition, developed through these classical works, also assumes a humanist and capacity-based conception of human reason and an instrumentalist view of technology (Schwarz 2021, 55).

These arguments show the shortcomings of the notion of (meaningful) human control formulated through capacity-based theories of agency. ‘Control’ refers to a unilateral relationship between two or more entities, with some entities influencing and having decision-making power over the others. However, when technologies meaningfully influence decision-making processes, in ways not determined by their users or designers, humans cannot meaningfully be in control. When decision-making is a product of the interaction between multiple entities, the notion of control cannot conceptualise the relationship between those entities.

### Negative impact of human–machine interactions on human ‘agential capacities’

Second, technologies can negatively impact humans by diminishing the cognitive skills required to exercise their ‘agential capacities’. Automation bias, for example, is a well-documented tendency of humans to over-rely on algorithmic decisions and ignore their own senses and other sources of information, even if they conflict with the algorithmic decisions (Zerilli et al. 2019, 561). Deskilling is another phenomenon in which human skills deteriorate over time due to prolonged interactions with and delegation of tasks to machines (ibid.). In such cases, human–machine interactions impact human abilities: the human who engages in interactions with machines becomes gradually less capable.

This shows that frameworks that assume the capacity-based approach fail to account for the empirical evidence concerning the dynamic and significant impacts of technologies on humans and the way humans exercise their ‘agential capacities’. Such initiatives may recommend having a human work alongside a machine to supervise automated decision-making and override the machine’s decisions when they are deemed morally or legally questionable. However, the human–machine pairing is ineffective when the human’s ability to perform these tasks is diminished *as a result of* working with a machine.

### Different capacities of the same entity leading to contrasting outcomes

The previous two problems of the capacity-based approach reveal a third, more fundamental problem of this approach. Governance ideas that are derived from a capacity-based account of agency assign roles to agents (e.g. that a human should be in-the-loop or on-the-loop) based on a selected set of capacities that they deem ‘agential’. This is based on the assumption that the interaction of entities with their surroundings is determined by ‘agential capacities’.

However, it is not true that a selected set of capacities of an entity would necessarily determine the interactions of the entity with its environment. Each entity has multiple capacities that would lead to different forms of interaction between the entity and its environment. In any given context, only some of these capacities are realised, but the capacities that are realised, are not necessarily ‘agential capacities’. For example, in any given situation, a human operator of a machine has the capacity to be on high alert and make reasonable interventions in the machine’s operation. But they also have the capacity to feel afraid, get tired, form biases or lose concentration or skill. In reality, actions and decisions of the operator may be determined by any (combination) of these capacities.

Moreover, regardless of how ‘agential capacities’ are defined, in practice it is impossible to separate them from other capacities of the entities that are endowed with them. Agential and non-agential capacities are capacities of the same entity (e.g. one and the same operator). It is impossible to have one, but not the other. Hence, assigning roles to human operators based on their ‘agential capacities’ may not result in a morally permissible or legally compliant performance. This is because there is no guarantee that ‘agential capacities’ would determine the kind of interaction that the human operator would have with the systems. In fact, considering empirical findings that demonstrate the tendencies of humans for automation bias, deskilling or alert fatigue, ‘non-agential capacities’ are very likely to govern human–machine interactions.

Arguments presented in this section demonstrate some shortcomings of the capacity-based approach for conceptualising human–machine interactions and as a theoretical basis for technology governance. The shortcomings of these governance ideas cannot be resolved by differently nuanced proposals that rely on similar assumptions about agency. *Any* proposal that assumes a capacity-based notion of agency will encounter similar problems. Thus, designing more advanced systems will not overcome the limitations of capacity-based theories. ‘Smarter AI’ or faster or more accurate systems will not solve these issues. The problem is not a problem of automation itself, nor the human operator itself, but rather how to design an efficient human–machine interaction (Berberian 2019, 119). As explained by Woodcock, to understand nuances of agency in human–machine interactions, we need to move away from rationalistic perspectives and instead examine how agency is manifested in practice (2024, 103).

## The relational approach

In the relational approach, agency is defined as an external and relational property of entities, not as their universal and intrinsic properties. Different notions of agency as a relational property have been developed, with each resting on a distinct metaphysics.

For example, according to Latour, agents are entities which ‘make some difference to the state of affairs’ (Latour 2007, 52–53); entities which make no difference and produce no transformations are not agents (ibid.). His metaphysical stance is that of the actor-network theory in which networks and their intra-relationships are given ontological primacy over actors within networks. Barad’s notion of agency is also relational. She defines agency as ‘intervening in the world’s becoming’ (Barad 2003, 824), emphasising that agency should not be understood as a property of entities, but as a property of *relationships*. Barad’s metaphysical idea is agential realism in which interactions ontologically precede objects, and objects derive their existence from interactions. According to Verbeek (2011) too agency is distributed over humans and non-humans. For him, agency is never purely human or purely technological; it rather is a product of human-technology *associations* (ibid., 53). Verbeek’s analysis is rooted in the post-phenomenological tradition, where technologically mediated relationships between the subject and the object are treated as the fundamental units of philosophical analysis.

Although each of the above relational notions of agency rests on a distinct metaphysics, they all consider agency to be a property of relationships or associations. None of these theories consider agency to be a property of any entity. They contend that when a combination of humans and technologies generates an outcome, agency is attributed only to the entire combination. This thesis can be dubbed as the ‘hybrid-agency thesis’.

I will evaluate the hybrid-agency thesis later in the paper. But before doing that, I conceptualise agency based on the notion of impact. The account of agency as impact will be derived from a pragmatist metaphysics and will be compatible with Coeckelbergh’s socio-relational approach in which agential considerations are based on social relations rather than capacities of entities in themselves (2010). However, while Coeckelbergh’s goal is to examine attribution of rights and moral worth to robots (and other entities, e.g. animals), my account of agency is intended to explain the dynamics of human–machine interactions and guide the governance of technical systems.

## Agency as impact

I propose conceptualising agency based on the notion of impact and defining agents as impactful entities. But first, I need to define what is meant by impact. Including all impactful entities as agents does not necessarily have explanatory benefits. A small asteroid may collide with, and hence ‘impact’, a planet outside the Milky Way. But nothing practically or normatively interesting may be achieved by ascribing agency to the asteroid. So, which instances of impact justify the attribution of agency, and which do not?

Two distinct theoretical routes can be taken to modify the link between impact and agency in order to avoid inclusion of *all* impactful entities as agents. One route is adopted by capacity-based theories. Capacity-based theories argue that we need to look *inside* impactful entities. They argue that only those impactful entities are agents that possess ‘agential capacities’. According to these theories, the asteroid of the example above would not be an agent because it does not have ‘agential capacities’. However, capacity-based theories include all entities that possess ‘agential capacities’ as agents, including those that do not necessarily have any actual impacts. For example, an alien hermit who spends their entire time contemplating the meaning of their solitary existence can be categorised as an agent, if they possess ‘agential capacities’. I do not further engage with capacity-based theories here and refer the reader to earlier discussions of the paper.

The second route, which I adopt, leads to a relational notion of agency. It averts from searching for intrinsic properties of impactful entities and instead turns to the relationship between instances of impact and the individuals who attribute agency to explain the events of social reality. Agency, in this sense, is not owned; it is attributed (Coeckelbergh 2018, 150). Whether an impactful entity is included in the domain of agents depends on the *reality* of the attributor.

‘Reality’ is here understood in a pragmatist sense. Reality is not a collection of entities that exist ‘out there’ independently of beliefs and practices of individuals. That would be a divine notion of existence that, as James argues, only concerns gods (James 1981, 924). Reality, rather, is shaped through our immediate experiences (Dewey 1905, 393). It includes things that an individual relates to; things that influence or play a role in an individual’s activities (Soltanzadeh 2022a, 143).

In this account, only those impacts that are experienced by and influence attributors lead to the identification of agency. The small asteroid that impacts a planet outside the Milky Way is not an agent because its impact is not experienced by and does not influence attributors (here: humans). It plays no role in any human activities. Similarly, an alien hermit residing on a remote planet would also not be considered an agent because the hermit is not present in the reality of human attributors. And this is regardless of the physical, sensory or cognitive capacities of the hermit.

Defining agency as impact results in a mind-dependent and subjective determination of agency: an agent is an agent because it is *perceived* as an agent. But this mind-dependence does not mean that anyone can decide on a whim whether they wish to include or exclude an entity in the category of agents. To be recognised as an agent, an entity needs to play roles in the activities, decision makings and actions of those who attribute agency. The entity must be practically real. So, although the determination of agency requires a subjective standpoint, the criterion for the determination of agency is not itself subjectively determined.

The alien hermit is an example of an entity that would be considered an agent by capacity-based accounts but would not be considered an agent in the account of agency as impact. However, since understanding agency as impact does not stipulate conditions for intrinsic properties that impactful entities need to possess to qualify as agents, any entity with any properties, regardless of being human or nonhuman, can potentially be an agent.

AI decision-support systems, which function as algorithmic equivalents of human advisors, should be considered agents. These systems gather, process and filter data to present to users to support their decisions. AI decision-support systems are used for production planning or for personalised purchase recommendations. They also assist experts in fields, such as medical practices, athletic performance analysis or military operations. When in use, these systems fall into the category of agents because they impact the beliefs, perceptions, intentions and actions of their human users, and they impact the operation of the socio-technical system to which they belong.

Since in this account, agents’ capacities are irrelevant for the attribution of agency, less complex objects can also impact attributors and become agents. A dice, for example, can be categorised as an agent when it is used for decision-making purposes, be it in the context of a board game or in real life (e.g. to decide what to wear, who to meet or where to go).

Nevertheless, it should be mentioned that when an entity does not have the capacity for a particular type of impact, it cannot make that impact. This is because, as discussed earlier, entities’ capacities influence the type of interactions that they can have with their surroundings. For example, a tree does not have the capacity to fly, a vacuum cleaner does not have the capacity to recommend chess moves and a human does not have the capacity to see in the dark without the use of tools such as flashlights or night vision goggles. But in any case, although there is a relationship between entities’ capacities and the interactions that they have with their surroundings, what constitutes agency is the latter, not the former.

So it is not the case that capacities are irrelevant for agency. Rather, only those capacities that lead to impacts should be considered relevant. Because each instance of impact may be linked to a different capacity, agency cannot be conceptualised by a universally defined set of capacities. This means that impact, agency, and relevant capacities for agency are context-dependent and dynamic. They are dynamic in the sense that an entity may be an agent in some contexts but not in others. This point can be explored a bit further.

An entity may lose its agential status for various reasons, such as being unused, incapacitated or broken. However, even if an entity is in use and fully functional, its agential status can change depending on the context in which it is used. A medical doctor, for example, may choose to rely on their own judgement and disregard the input from an ultrasound system when diagnosing a patient’s medical condition. The doctor’s judgement may of course be formed through other means and technologies, such as blood tests and X-rays. But in this case, the ultrasound technology would not be considered an agent as it is not impacting the doctor’s decision-making. But the same patient may visit a second doctor who relies on the ultrasound images for diagnosis. In this latter case, the ultrasound technology would be impactful and hence will gain agential status. Even though in both cases what the ultrasound technology produces is the same, its agential status would be different. Hence, the agential status of an entity cannot be determined without considering the context of use.

This point also applies to humans. A person may perform similarly in different contexts, but their performance may be ignored in one context and have significant impact in another. For example, a technician’s ideas about responsible product development may be disregarded in one company but taken seriously in another. Because the technician’s impact is different in each company, their agential status would also be different.

## Levels of agency

The account of agency as impact is relational, but it differs from most existing relational theories because in this account, agency is not *attributed to*, but *requires* relationships. In this section, I elaborate this point to demonstrate the advantages of this account of agency for technology governance purposes. By doing so, I also explicate the notion of ‘levels of agency’.

As discussed earlier, many current relational theories agree on the hybrid-agency thesis, which states that agency is not a property of any entity, but rather a property of relationships and associations of entities. In the context of socio-technical systems, this means that agency is distributed over human-technology hybrids, rather than attributed to any single component. No entity within the hybrid can be considered an agent on its own.

The hybrid-agency thesis, which refrains from attributing agency to any elements within hybrids, faces some problems. First, it is difficult to apply this thesis to practical cases which are concerned with regulatory and social implications of agency. In particular, human agency is fundamental to the domain of law which seeks to define and attribute liability for wrongdoings to individuals who are responsible for the outcomes of their actions. The idea that agency can only be attributed to hybrids would not be pragmatically useful for holding individuals accountable for their wrongdoing.

Moreover, the hybrid-agency thesis encounters a significant conceptual challenge in determining how the line between what is within and what is outside a hybrid is drawn. Since any small hybrid may be a part of a bigger hybrid, under the hybrid-agency thesis, any local analysis of agency may result in examining an overly inflated and complex socio-technical hybrid. Consider a military operator who is using a radar system to detect incoming enemy missiles. According to the hybrid-agency thesis, agency is not a property of the operator or the radar system; rather, agency is a property of the radar-operator hybrid as a whole. But imagine that the radar-operator hybrid is a part of a bigger hybrid, say a military unit. And the commander of the unit may be responsible for receiving data from the radar-operator hybrid and using an anti-missile system to intercept incoming missiles. This bigger hybrid would include the initial operator and radar system, the commander and an anti-missile system. According to the hybrid-agency thesis, the only agent in this bigger hybrid is the military unit as a whole; nothing *within* the military unit, including the initially identified, radar-operator hybrid, should be considered an agent. Now imagine that the bigger hybrid (i.e. the military unit) is a part of an entire military, which also includes many other units. Once again, the hybrid-agency thesis would imply that agency is distributed over the whole military: nothing within the entire military (including the radar-operator hybrid or the military unit) can be considered an agent. This argument can be extended even further to include allied forces and other states. Each time a larger hybrid is identified, the hybrid-only thesis would consider agency to be a property of the larger hybrid only, and nothing within the larger hybrid can be an agent. This demonstrates that the hybrid-agency thesis is not a pragmatically helpful thesis, since not much can be achieved by restricting the attribution of agency to extremely large socio-technical systems.

The problem of over-sized and practically unhelpful attributions of agency can be addressed by recognising that agency can be manifested at different *levels*. Assigning agency to large socio-technical systems should not preclude the possibility of assigning agency to smaller hybrids within those systems. Similarly, assigning agency to smaller hybrids should not preclude the possibility of assigning agency to individual components within those hybrids. Agency can be attributed and studied at various levels, from the low levels of individual entities to the high levels of socio-technical systems. The unifying concept in attribution of agency at different levels is that of impact. Any entity, whether it is a hybrid or an individual human or technology, can be categorised as an agent if it has an impact on other entities.

In the previous example, the radar system is an agent as it impacts the perception and situational awareness of the operator. The radar-operator hybrid is an agent because it impacts the beliefs of the commander about incoming missiles. The commander, in turn, is an agent for making the decision to use the anti-missile system. The entire unit is an agent in carrying out the task of detecting and shooting down incoming missiles. This task also impacts the tactics and performance of other units in the military as well as those of allied and opposing forces. This means that a hybrid can be scaled down or up. When scaling up, the impacts of bigger and more complex hybrids are studied; when scaling down, the impacts of smaller units that constitute the hybrid are examined.

Higher levels of agency can be observed and studied by spatial or temporal expansion of hybrids. The previous example illustrates a spatial expansion of the air-defence hybrid to include a larger number of entities whose impact can be studied in the same temporal context. However, hybrids can also be expanded temporally, allowing for examination of past events that contributed to the current operation of a hybrid. For instance, in the case of large military or medical socio-technical systems, the impact of designers and regulators on the functioning of the hybrid can be examined by looking back at their past actions. Although designers and regulators work in different temporal (as well as spatial) contexts, expanding the hybrid allows for the study of their impact. This point will be further explored in the next section through the idea of ‘channels of impact’.

## Channels of impact and degrees of agency

The impact of different entities on the performance and outcomes of the hybrid to which they belong is not the same. Some entities may have a greater impact on the performance and outcomes of the hybrid than others. In the account of agency as impact, degrees of impact translate into degrees of agency. So, here, agency is not a binary concept, but can be manifested on a spectrum, with more impactful entities having higher degrees of agency.

Imagine a group of people planning to visit multiple tourist sites in a holiday town. They may discuss among themselves the most efficient way to reach different points of interest. They may also use online maps and frequently ask locals for advice. But it might be that one of the group members is a bit authoritarian and often convinces the others to trust their intuitions and follow their lead. In this case, it can be argued that this person has a greater impact on the group’s decision-making processes and has a higher degree of agency than others.

However, determining degrees of agency is not always as straightforward as in the example above, where all entities involved impact the group *in a similar way*, i.e. by suggesting routes between different locations. Understanding degrees of agency in socio-technical systems is usually more complex because different entities of a socio-technical system have different functions and impact the system *in different ways*. In complex socio-technical systems, determining degrees of agency requires identifying *channels of impacts*.

### Channels of impact in the context of use

There are six general channels through which entities of different kinds, including hybrids, can have an impact on their surroundings. These channels can be referred to as ‘channels of impact’ and include (1) goal setting, (2) sensation, (3) evaluation, (4) action, (5) design and (6) regulation.

The first channel of impact is *goal setting*. An entity can impact the operation of a hybrid by forming intentions for the hybrid to perform certain actions and achieve certain outcomes. For example, a doctor may decide that a patient needs to undergo surgery, or a military commander may form the intention that an incoming aerial object should be targeted and destroyed. Even if they do not perform any other actions, through their decisions and intentions, and via mediums such as written instructions, referral letters or direct orders, the doctor and the commander impact the operation of the hybrids to which they belong.

However, goals are not set in a vacuum. They are a response to what is perceived and what is desired to be the case. The first step for setting goals is gathering information. Information is often gathered via sensors or sensory inputs and is often dependent on technological possibilities. Radar systems and medical screening devices, together with their operators, collect information and present it to other elements of the hybrid. Through the channel of *sensation*, they impact the hybrid’s perception of the world, which in turn influences the actions taken by other elements of the hybrid.

The third way in which entities can have an impact is through the channel of *evaluation*, which connects sensation to goal setting. Raw information is not useful for goal setting unless it is processed and evaluated. Processing and evaluating information can be achieved either by human experts, algorithms or a hybrid of humans and nonhumans. A doctor examining medical tests to diagnose a patient’s condition plays this role. Similarly, AI systems can also process and evaluate certain types of information, such as identifying cancer cells or incoming aerial threats. These would in turn impact the operation of the rest of the hybrid through influencing decision-making processes, and the intentions which are subsequently formed.

The fourth channel of impact is the channel of *action*. Here, action is used in its broad sense and does not require the presence of intentional mental states. In this broad sense, entities of different kinds, including technical systems, can act by causing changes to the state of affairs. A missile that destroys incoming aerial threats and a surgeon who performs an operation on a patient impact other entities or hybrids through the channel of action.

There is a reciprocal relationship between action and other channels of impact. On the one hand, none of the other channels of impact would be effective unless some entities take action to make changes to the outside world. For example, it is not sufficient for a medical team to evaluate a patient’s medical tests and merely decide that the patient needs surgery, and leave it at that. Someone also needs to actually perform the surgery and impact the patient’s state. So, although some channels of impact may not be present in some hybrids, the channel of action, in some shape or form, is always necessary for the operation of a hybrid. On the other hand, actions always need to be understood in relation to the intended goals. Two similar actions can have different meanings and impacts depending on what is believed to be the purpose of performing them (Soltanzadeh 2022b, 281). Cutting through someone’s skin and detonating an object, for instance, can have vastly different meanings and impacts depending on the decision-making context.

Sensation, evaluation, goal setting and action are channels of impact within the context of use. They signify four ways in which entities of different kinds can influence the operation of socio-technical systems.

An entity within a socio-technical system can itself have multiple functions and make impacts through various channels. For example, the individual who evaluates medical test results and determines that a patient should undergo surgery may be the same person who performs surgery. Moreover, given that the word ‘entity’ is used in its broad sense here, an entity within a socio-technical system impacting other entities through a specific channel may itself be a hybrid. For example, a report detailing the medical test results of a patient impacts the rest of a medical hybrid through the channel of sensation. However, the report may itself be generated by a group of smaller hybrids, such as the one that generates blood test results, one that produces ultrasound images and one that prepares the report and sends it for decision-making.

### Design and regulation

The first four channels of impact, i.e. goal setting, sensation, evaluation and action, are present in the context of use. To identify the fifth and sixth channels, the level of analysis of a socio-technical system needs to be temporally and spatially expanded.

The fifth channel of impact is design, understood broadly to include the engineering and manufacturing of technical products. The channel of design is important for various reasons. First, the impact of a hybrid on its surroundings is conditioned by available technical systems: what users can achieve in different circumstances depends on the technical possibilities available to them. Second, design impacts the relationship between entities present in the context of use: depending on the design of a technical system, different entities need to interact in particular ways with each other for the system to function. In particular, the design features of technical systems determine the extent and ways in which operators need to interact with the systems.

The design of a technical system can impact the consequences of its use, particularly when users assign the same function to a system that designers intended. And this is often the case with complex systems. Simple tools and small handheld artefacts may be used for various purposes that are unpredicted by designers. However, users often assign the same function to large or technically complex machines as the designers intended.

When users use technical systems for the purpose intended by designers, an epistemic route can be drawn between the users’ beliefs and the designers’ intentions. Users follow the instructions or ‘use-plans’ provided by the designers (Houkes and Vermaas 2004, 52–71), which they learn through means such as meetings, manuals or workshops. These instructions create a framework for users to justify their beliefs about how a technical system can be used and what can be achieved by using it.

However, even when technical systems are deployed according to the designers’ instructions, they may bring unintended or unforeseeable consequences. And this is where the sixth channel of impact, *regulation*, becomes crucial. Regulation is achieved through deliberative norm-setting processes, where ‘norm’ encompasses a wide range of social, cultural, moral values, regulatory and ethical principles, and domestic and international laws. Norms influence the operation of socio-technical systems by affecting decision-making processes.

Not all norms are explicitly defined or communicated. Cultural expectations and social and moral values, for example, can also impact decision making. However, similar to emotions, their influences do not go through deliberative processes. While their impact should be acknowledged, understanding how cultural expectations, social and moral values or emotions impact the formation and operation of socio-technical systems requires an empirical study which is outside the scope of this paper.

But norms can also be set through deliberative processes. Deliberative norm setting can be established by actors such as managers, medical supervisors or military commanders who set out rules and codes that may not be legally binding, but still influence the operation of socio-technical systems. *Binding* legal norms, on the other hand, are found in formal sources, such as domestic legislation or international treaties. In societies that are governed by the rule of law, these norms have a high degree of impact. They impose legal obligations that guide conduct and decision making.

One of the key ways in which regulation influences the operation of socio-technical systems is through holding wrongdoers accountable. Many individuals and groups take their legal responsibilities seriously. So, holding them responsible can influence decision-making processes and deter criminal actions. However, the extent to which legal responsibility can be attributed to different entities depends on the availability of effective mechanisms. Absence of effective frameworks to hold individuals or collectives responsible for violating the law results in a diminished degree of impact of the channel of regulation. This, in turn, can lead to arbitrary uses of technical systems without any sense of accountability. Moreover, the presence of frameworks for attributing responsibility does not necessarily make the channel of regulation very impactful. Higher degrees of impact of the channel of regulation can be obtained when responsibility attributions are supported by enforcement mechanisms.

So, design and regulation shape the physical and social conditions for the operation of socio-technical systems in the context of use. While design and regulation operate in spatially and temporally distant contexts, high degrees of agency can be exercised through these channels.

In this way, understanding the channels of impact can provide a more nuanced conceptualisation of degrees of agency. An important note here is that an entity’s degree of agency cannot be solely determined by the amount of time they spend performing their tasks. The channel of impact also plays a role in determining degrees of agency. Channels of impact are not equally impactful.

Traditionally, entities whose impacts come through the channel of goal setting are considered to be more impactful than others. An entity that sets the goals of the hybrid determines the role of other entities in the hybrid. The high degree of impact of the channel of goal setting is also reflected in the role of intent in assigning criminal responsibility. For example, a commander of an air defence unit who orders a soldier to shoot down an aerial object has a higher degree of agency than the soldier or the weapon. The hybrid’s intention to destroy the aerial object is set by the commander.

However, one of the influences of AI and modern technologies is that they problematise this traditional hierarchy by making the channels of sensation and evaluation potentially more impactful than the channel of goal setting. AI systems used for medical diagnosis or military target identification may have higher degrees of agency than the specialist doctors or military commanders. Even though the specialists and commanders may be the ultimate decision makers, if they solely rely on the systems’ inputs and simply follow the systems’ diagnoses or recommendations, they would have lower degrees of impact and agency than the technical systems.

## Conclusion

Intricacies of human–machine interactions cannot be explained through the capacity-based approach to agency, according to which, agency is defined based on intrinsic ‘agential capacities’. Hence, capacity-based assumptions about agency should not guide the governance of technical systems, especially AI-enabled machines. Capacity-based theories dismiss the fact that agency can emerge as a product of interactions. They give too much explanatory weight to ‘agential capacities’ and simplistically assume that these capacities determine the interaction of humans with machines. They disregard the fact that each entity has multiple capacities that, depending on contextual factors, can lead to contrasting outcomes. Since the problem of capacity-based governance initiatives is their underlying assumption about agency, any other initiative that shares this assumption would be equally inadequate.

‘Agency as impact’ is an account of agency that relies on empirical evidence and has explanatory and pragmatic advantages. On the one hand, this account of agency is successful in explaining the dynamics of human–machine interactions by taking into account documented phenomena, such as deskilling, automation bias and alert fatigue. And on the other hand, it can serve the function of creating a metaphysical foundation for the governance of technology. This account of agency shifts the discussion away from making essentialist distinctions between agents and non-agents. Instead, it focuses on the context-dependent and dynamic analysis of the levels, degrees and channels of agency.

Each channel of impact provides a distinct way for entities to exercise their agency. But channels of impact are not equally influential. Traditionally, goal setting through forming intentions has been considered the most influential channel of impact. However, with the rise of AI decision-support systems, the channel of evaluation has become more impactful and can lead to higher degrees of agency.

While this paper establishes a metaphysical foundation for agency in the governance of technology, it inevitably leaves some issues unaddressed, both on the practical and theoretical fronts. For example, the analysis of degrees of agency can be used to conceptualise degrees of *human* agency. Given that many AI governance frameworks are concerned with the role of humans, it is valuable to explore degrees of human agency in each channel of impact and in the overall operation of socio-technical systems. Design and regulation are two channels of impact that can be particularly relevant in this respect. While technical systems may have high degrees of agency in the context of use, high degrees of human agency can be exercised through design and regulation. However, investigations of degrees of human agency should be undertaken with caution to avoid falling back to humanistic capacity-based conceptions of agency. Another related topic is that of responsibility. In both moral philosophy and law, strong connections are traditionally drawn between the categories of agents and responsibility-bearers. This paper can support those works that challenge the extensional identity between these two categories. However, determining whether responsibility-bearers constitute a subcategory of agents or if there are no necessary connections between these two categories requires further research.

## Data Availability

I do not analyse or generate any datasets, because my work proceeds within a theoretical and conceptual approach.
